# Carbon:Nitrogen Ratio Affects Differentially the Poly-β-hydroxybutyrate Synthesis in *Bacillus thuringiensis* Isolates from México

**DOI:** 10.3390/polym17141978

**Published:** 2025-07-18

**Authors:** Marco Tulio Romero Sanchez, Shirlley Elizabeth Martínez Tolibia, Laura Jeannette García Barrera, Pavel Sierra Martínez, Jorge Noel Gracida Rodríguez, Valentín López Gayou, Víctor Eric López y López

**Affiliations:** 1 Centro de Investigación en Biotecnología Aplicada, Instituto Politécnico Nacional, Santa Inés Tecuexcomac 90700, Tlaxcala, Mexico; romero.sanchez.marco.tulio@gmail.com (M.T.R.S.); lgarciab@ipn.mx (L.J.G.B.); vlopezg@ipn.mx (V.L.G.); 2 Instituto de Investigaciones en Materiales, Universidad Nacional Autónoma de México, Mexico City 04510, Mexico; smartinezt@materiales.unam.mx; 3 Centro de Investigación Especializada en Microbiología, FCQB, Universidad Autónoma de Guerrero, Av. Lázaro Cárdenas s/n, La Haciendita, Ciudad Universitaria Sur, Chilpancingo 39087, Guerrero, Mexico; pavelsierra@uagro.mx; 4 Facultad de Química, Universidad Autónoma de Querétaro, Cerro de las Campanas, Querétaro 76010, Querétaro, Mexico; gracidaj@netscape.net

**Keywords:** biopolymers, fermentation, P(3HB), *Bacillus thuringiensis*

## Abstract

Poly-β-hydroxybutyrate (P(3HB)) represents a suitable alternative for plastic replacement, since it consists of intracellularly produced polyesters by different microorganisms including *Bacillus thuringiensis* (Bt). P(3HB) conserves most of the properties of petroleum-derived plastics; however, some drawbacks are the production costs, processing times, and bioseparation techniques, limiting its extended use. Bt has production advantages over other microorganisms, such as those growing in conventional or non-conventional substrates, with short periods of fermentation, which make it an interesting candidate to develop optimized production processes. In this work, we identified P(3HB) producers from 72 isolates of Bt, from which we selected four potential candidates. These isolates were cultivated under different carbon:nitrogen (C:N) ratios of 3, 7, 30, and 50 in a complex medium named (CM). Here, the best conditions for growth in Bt isolates were C:N 3 and 7 ratios, whereas for P(3HB) production they were C:N 7 and 30. Following this, an experiment in a bioreactor was conducted with isolate 81C with the selected C:N ratio of 30, where the produced P(3HB) achieved a maximum at 10 h. Fourier transform infrared spectroscopy (FTIR)was used to characterize flask and bioreactor cultures. It must be mentioned that although a higher concentration of medium was used, this did not improve P(3HB) accumulation. This research demonstrates that C:N ratios can differentially influence growth and P(3HB) accumulation in Bt isolates, which can serve as a reference to develop P(3HB) production processes using Bt as a microbial production platform.

## 1. Introduction

Petroleum-derived plastics are currently one of the main pollution sources, and most of them are resistant to degradation, which leads to their accumulation causing damage to different ecosystems around the world [1]. Plastics normally take between 20 and 100 years to degrade into soluble monomers, causing a range of issues related to environmental, water, and air pollution, also generating toxic gases during their decomposition process [2,3]. In this regard, it is estimated that by 2050 near 12,000 Mt of non-biodegradable polymer waste will be landfilled [4]. Some alternative solutions have been provided from biotechnology, with the implementation of bioprocesses using efficient microbial production platforms, where high-value products such as biofuels, enzymes, pharmaceuticals, and biopolymers have been obtained [5]. Therefore, it is essential to implement environmentally friendly alternatives such as the use of polyhydroxyalkanoates (PHAs), a family of non-toxic, biodegradable biopolymers with mechanical, physical and thermal properties similar to those of fossil plastics such as polypropylene and polyethylene. One of the most studied biopolymers is poly(3-hydroxybutyrate) (P(3HB)) which accumulates intracellularly in the form of granules, when there is an excess of a carbon source and a limitation of nitrogen, phosphorus, magnesium, and potassium [6]. Currently, the commercial production of PHA is carried out from cultures with Gram-negative bacteria such as *Pseudomonas*, *Escherichia coli*, and *Cupriavidus necator*. The latter is the most commonly used bacteria in industry for the production of P(3HB), but the major drawback is the long time it takes to complete biopolymer synthesis, exceeding 48 h [3,7]. On the other hand, *Pseudomonas* and *E. coli* produce endotoxins, in addition to the lipopolysaccharides that are also released during the extraction processes of the biopolymers, thus, affecting their purification and quality [8]. On the contrary, Gram-positive bacteria such as *Bacillus* have been widely studied to produce PHA using various agro-industrial residues, with the advantage of lacking lipopolysaccharides, which reduces purification costs which represents one of the main challenges for the industrial production of PHA [9,10]. In addition to its mechanical and physical properties, P(3HB) has been determined as a biocompatible material, allowing applications focused in medicine for the development of scaffolds for treating bone abnormalities and microspheres, for the controlled release of antithrombotic drugs like dipyridamole [11,12]. With the advent of novel food industry technologies, P(3HB) films have been explored as possible alternatives to traditional plastics, for the packaging of fat-based products such as mayonnaise and cheese [3]. Despite this, the production of biodegradable plastics for single use applications remains one of the greatest challenges to be solved. Therefore, in this work we selected four P(3HB) producer strains from 72 Bt isolates using different C:N ratios in the CM culture media. A comparison of cultures at flask and bioreactor level is presented, as well as the characterization of the extracted P(3HB). This information could contribute to establishing novel strategies for developmental processes applied to P(3HB) production, using Bt as a production platform.

## 2. Materials and Methods

### 2.1. Bacillus thuringiensis Isolates

Bacterial isolates were obtained from one kilogram of soil collected from areas with stagnant water, where traces of accumulated rainwater were evident. Samples were collected at the coast of Guerrero, Mexico, and stored in polyethylene bags at room temperature (28–32 °C) until arrival at the laboratory, where they were kept at 4 °C until further processing. One hundred grams of each sampled soil was resuspended in 100 mL of sterile distilled water, placed in a water bath with boiling for 10 min. Subsequently, 1 mL of supernatant was used to perform serial dilutions, which were plated on nutrient agar. After 5 days of incubation at 30 °C, colonies exhibiting a waxy appearance and consistent morphology with the *Bacillus* genus were selected for microscopic examination, using an oil immersion lens to detect the presence of spores and parasporal crystals. Moreover, isolates were stained with malachite green to confirm the presence of spores and toxin crystals.

Seventy-two bacterial isolates from beach ecosystems in Guerrero, México, were previously identified and categorized as *Bacillus thuringiensis* species. The obtained spores from isolates were preserved on sterile paper disks at a concentration of 10^7^ spores per mL and preserved at 4 °C. All isolates were grown in Luria Bertani (LB) liquid medium (1% casein peptone, 1% NaCl, 0.5%) and incubated at 30 °C, for 4 h at 200 rpm in a New Brunswick Scientific model 4220 incubator (Edison, NJ, USA). They were then inoculated into peptonized milk medium (10 g casein peptone, 2 g dextrose, 0.02 g FeSO_4_·7H_2_O, 0.02 g MgSO_4_·7H_2_O, 0.002 g ZnSO_4_·7H_2_O, 0.002 g MnSO_4_·7H_2_O, 0.3 g yeast extract in a final volume of 1 L) until the end of sporulation at 72 h (>95% free spores in the medium) and monitored by phase contrast microscopy.

### 2.2. Selection of P(3HB) Producers Using Sudan Black Dye

To confirm the presence of P(3HB) granules, bacteria isolates were incubated on mineral medium plates containing the following (g/L): glucose (7), NH_4_Cl (0.1), MgSO·7H_2_O (0.2), CaCl_2_ (0.01), KH_2_PO_4_ (1.5), (NH_4_)_2_Fe(SO_4_)_2_∙6H_2_O (0.06), for 48 h at 30 °C and pH = 7. A sample was taken and fixed on a slide by adding an ethanolic solution with Sudan black (0.05%), followed by incubation for 15 min and rinsing with 96% ethanol, to remove excess dye [13,14]. Safranin was then added for one minute to create contrast, and the samples were washed with distilled water. The slides were observed under a microscope at 100× (Primo Star Carl Zeiss, Göttingen Germany).

### 2.3. Molecular Identification of Selected Bacillus thuringiensis Isolates

The molecular identification of the selected P(3HB) producing isolates was performed by genomic analysis, where DNA was extracted from bacteria using a bacterial genomic DNA isolation kit (Puregene Cell Core Kit, QIAGEN, Hilden, Germany), as described in the protocol for Gram+ bacteria. The extracted DNA was used as a template to amplify the 16S rRNA gene by polymerase chain reaction (PCR), using previously reported conditions [15]. PCR products were purified using the Zymo DNA Clean & Concentrator Kit (Zymo Research, Tustin, CA, USA), according to the supplier’s instructions. The purified PCR products were sequenced at the National Laboratory for Agricultural, Medical and Environmental Biotechnology (LANBAMA-IPICYT). Sequence analysis was performed using Geneious Prime^®^ 2023.0.4 analysis software, and results were uploaded to GenBank for accession numbers. In addition, a phylogenetic tree was constructed using MEGA 10.1.8 to assess the evolutionary relations among the isolates.

### 2.4. phaC Gene Amplification

**Figure 1:**
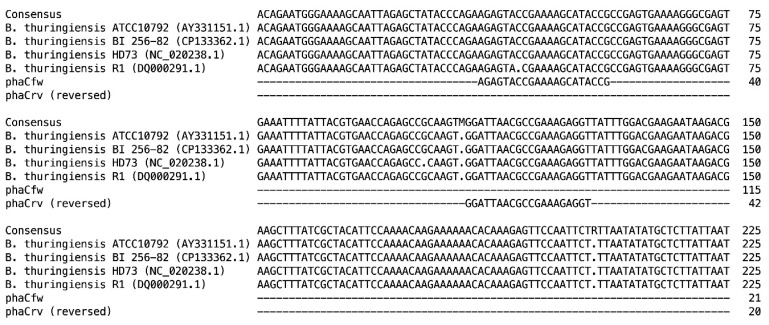
Location of forward and reverse primers on *phaC* gene from Bt isolates.

To confirm the presence of P(3HB) producer isolates, PCR amplification was performed using the primer pairs Forward: 5′AGAGTACCGAAAATACCG3′ and Reverse: 5′ACCTCTTTCGGCGTTAATCC3′ for the amplification of the *phaC* gene, which encodes P(3HB) synthase [9]. The primer design was based on the nucleotide sequences from Bt AY331151.1; CP13362.1; NC_020238.1 and DQ000291.1, obtained from the National Center for Biotechnology Information (NCBI), as shown in Figure 1. The PCR amplification conditions were as follows: (1) initial cycle at 95 °C for 3 min, (2) 35 cycles of denaturation at 95 °C for 1 min, annealing at 57.9 °C for 45 seg, and extension at 72 °C for 1 min, and (3) a final extension performed at 72 °C for 10 min. PCR products were visualized by gel electrophoresis (1.5% agarose).

### 2.5. Evaluation of C:N Ratio for P(3HB) Production During Flask and Bioreactor Cultures

The culture medium used for P(3HB) production was the clean medium (CM) [16] at different C:N ratios 3, 7, 30, and 50. It is noteworthy that CM has been used in our research group for different purposes in *B. thuringiensis* cultivation [16,17,18,19,20].

C:N is defined as the ratio of carbon (C) content derived from glucose in the medium, to nitrogen (N) content derived from soy peptone and yeast extract, as shown in Table 1. Flask cultures were carried out in a diluted version of CM to avoid oxygen limitation, while the bioreactor culture medium was the same composition as CM [16] with variations in the concentration of glucose 22.5 g/L, soybean peptone 1.5 g/L, and yeast extract 1.8 g/L. The mineral composition added to all cultures was as follows: 3 g/L KCl, 0.2 g/L MgSO_4_·7H_2_O, 40 mg/L MnSO_4_, 30 mg/L CoCl_2_, 7.5 mg/L CuSO_4_·5H_2_O, 5.8 mg/L ZnSO_4_·7H_2_O, and 1.35 mg/L FeSO_4_. Flask cultures were carried out in 1 L Erlenmeyer flasks containing 190 mL of CM at 200 rpm, pH 7, and 30 °C. Inoculum cultures were prepared by adding spore disks of each isolate in CM media (C:N 7), incubated in a shaking incubator at 30 °C, until the exponential phase was reached. Then, 10 mL of each culture was used to inoculate fresh medium at the different C:N ratios. These experiments were conducted in triplicate. Batch fermentation was carried out in a 7 L bioreactor (BIOSTAT^®^ A plus, Sartorius, Göttingen, Germany) with 4 L operational volume. A log-phase inoculum was prepared from a spore disk through one stage culture in CM and seeded to the bioreactor at a ratio of 5% *v*/*v*. The operating conditions of the bioreactor were as follows: 4 L working volume, 30 ± 1 °C, 600 rpm stir and an aeration rate of 1 vvm (volume of air per volume of medium per min). The pH was automatically controlled at 7 using 1M NaOH and 1M H_3_PO_4_. Fermentation samples were taken periodically for analysis.

### 2.6. Analytical Methods

Glucose was measured with a biochemical analyzer (YSI 2700 Select, Yellow Springs, OH, USA) every 2 h during the fermentation lapse (24 h). Bacilli were counted in duplicate in a Neubauer chamber, and dilutions were made to avoid cell clumping. The sporulation efficiency was calculated by dividing the maximum spore count by the maximum bacillus count and multiplying the quotient by 100. The specific growth rate (*µ*) was calculated by adjusting the experimental biomass data to an exponential model:

(1) $$X_{t}=X_{0}e^{µt}$$

where *X_t_* is biomass (cells/mL) at any time of fermentation, *X*_0_ is initial biomass (cells/mL), *t* is time (h), and *μ* is specific growth rate (h^−1^). Cell dry weight (cdw) was estimated for maximal cell counts with the relation of Rodriguez Monroy and de la Torre (1996) considering a single cell weight to be 2.3 pg [21].

### 2.7. P(3HB) Quantification by UV Spectrophotometry

For the P(3HB) quantification from flask and bioreactor cultures, 100 µL of culture samples at different times were centrifuged at 10,000 rpm for 5 min to obtain pellets. Cells were washed with PBS buffer (pH 7.4), centrifuged, resuspended with glycine–HCl buffer (0.1 M), and subsequently centrifuged at 10,000 rpm for 5 min. The cellular pellets were resuspended in PBS and then incubated for 2 h at room temperature. Later, samples were centrifuged at 12,000 rpm and resuspended in 1 mL chloroform. The separated phase was transferred to glass tubes and placed in a water bath at 50 °C until chloroform was evaporated. Then, 1 mL of H_2_SO_4_ was added and boiled for 10 min. The absorbance was measured in a UV spectrophotometer at 230 nm. To estimate the concentrations, a calibration curve was prepared in duplicate using a P(3HB) standard (Sigma-Aldrich, Hamburg, Germany) with a concentration range between 20 and 200 µg/mL. Concentration was correlated with the absorbance, and the equation was obtained from the calibration curve [22].

### 2.8. P(3HB) Extraction and Characterization by FOURIER Transform Infrared Spectroscopy (FTIR)

Samples of 100 mL were recovered at the end of the flask and bioreactor fermentations and then centrifuged at 6000 rpm for 20 min. Supernatants were discarded and cell pellets were recovered and washed with sterile distilled water. Cells were resuspended with 35 mL of 5% sodium hypochlorite and 35 mL of chloroform. The mixtures were placed in a shaking incubator at 200 rpm and 35 °C for 2 h. The suspension was subsequently transferred to a separatory funnel and incubated statically for 30 min, to allow phase separation. The upper phase contained the hypochlorite solution, whereas the intermediate phase showed cell debris, and the lower phase contained P(3HB) solubilized in chloroform. The lower phase was therefore decanted into a beaker, and two parts of methanol were added to precipitate P(3HB), with subsequent drying by evaporation at 60 °C to obtain a white powder [23]. The obtained P(3HB) powder was compared with the commercial P(3HB) standard (Sigma Aldrich) by FTIR spectroscopic characterization on a Bruker Vertex FTIR spectrometer (Bruker Optics Corporation, Billerica, MA, USA), to obtain the characteristic spectra from 400 to 3500 cm^−1^ [24].

## 3. Results and Discussion

### 3.1. Selection and Identification of P(3HB) Producer Isolates by Sudan Black Dye

**Figure 2:**
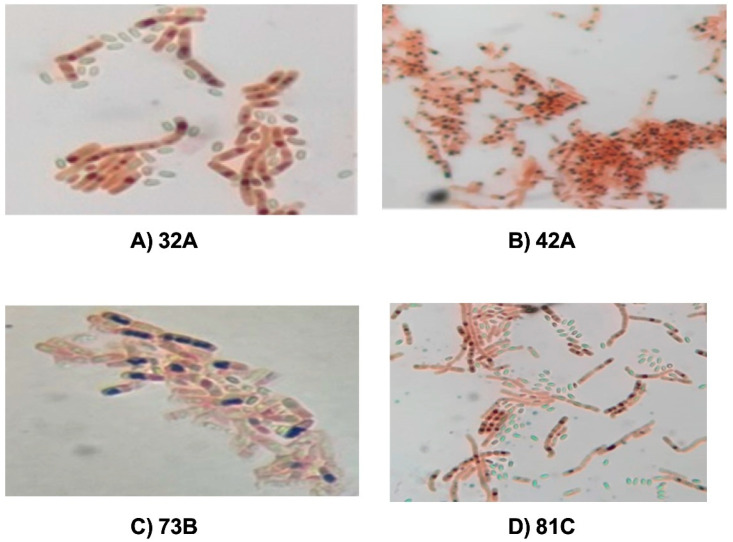
Microscope visualization of P(3HB) granules stained by Sudan black at 100× from *B. thuringiensis* selected isolates. (**A**) 32A, (**B**) 42A, (**C**) 73B, and (**D**) 81C.

Seventy-two isolates from soil samples from Guerrero, México were reactivated. These microorganisms were characterized by their colonial morphology and the observation of crystals by phase contrast microscopy. The 72 isolates were confirmed to have the presence of Bt-crystals, being evaluated as well for P(3HB) accumulation by Sudan black staining. The Bt isolates coded as 32A, 42A, 73B, and 81C presented the largest visible dark blue granules in their cytoplasm, when visualized under a microscope with a 100× objective, as shown in Figure 2. These results were considered positive evidence of P(3HB) accumulation, since the Sudan black dye has a strong affinity for the lipids present in the P(3HB)-containing granules, and the microscopic results partially revealed that the intracellular content of lipid and biopolymer is associated with the cytoplasmic materials [25,26]. Therefore, isolates 32A, 42A, 73B, and 81C were used for further experiments.

### 3.2. Bacillus thuringiensis Identification by 16S rRNA Gene Sequence and Identification of phaC Gene

**Figure 3:**
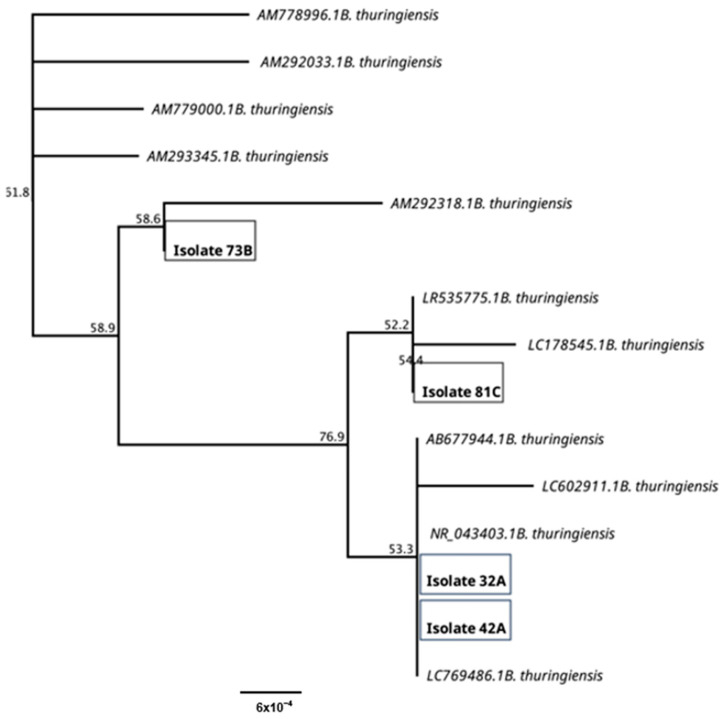
Phylogenetic analysis from Bt P(3HB) producer isolates 32A, 42A, 73B, 81C and *Bacillus thurgiensis* sequences previously published.

DNA was extracted from Bt isolates for 16S rRNA gene amplification to construct a phylogenetic tree from 11 Bt 16S rRNA gene sequences obtained from the NCBI, as shown in Figure 3. Sequences were analyzed using Geneious Prime^®^ 2023.0.4 analysis software, showing a high similarity (100%) with *Bacillus thuringiensis,* for all isolates presented in this work. The gene sequences obtained from the P(3HB) producing isolates were deposited in GenBank under accession numbers 32A (PQ656802.1), 42A (PQ656803.1),73B (PQ656804.1), and 81C (PQ656805.1).

**Figure 4:**
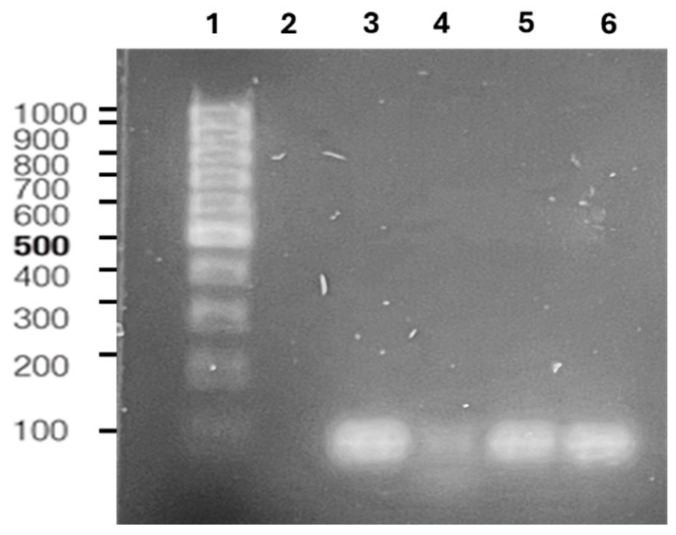
*PhaC* amplification from P(3HB) producer isolates: (1) 1000 pb molecular weight marker; (2) negative control; (3) 32A; (4) 42A; (5) 73B; (6) 81C.

Bt is a microorganism capable of producing P(3HB), therefore molecular studies were performed to identify the *phaC* gene which encodes the P(3HB) synthase PhaC. This enzyme is crucial for PHAs biosynthesis, as it facilitates the incorporation of different hydroxyacyl-CoAs (HA-CoAs) into the elongating PHA chains [27]. Accordingly, gene amplification by PCR was performed in this region to verify the presence of this gene in the P(3HB) producing isolates. The amplification shows a 98 bp length fragment corresponding to this gene, as observed in Figure 4. To complement these results, the extraction of the biopolymers produced by Bt isolates was further analyzed by FTIR. These results allowed us to determine that 32A, 42A, 73B, and 81C isolates have the potential to synthesize P(3HB), and belong to the *Bacillus thuringiensis* group, which has been reported to have this property. Furthermore, complementing our previous results with the *phaC* amplification served to confirm that the selected Bt isolates are capable of producing P(3HB) under specific conditions, as described below.

### 3.3. Effect of Different C:N Ratios on Growth and P(3HB) Accumulation in B. thuringiensis Isolates

**Figure 5:**
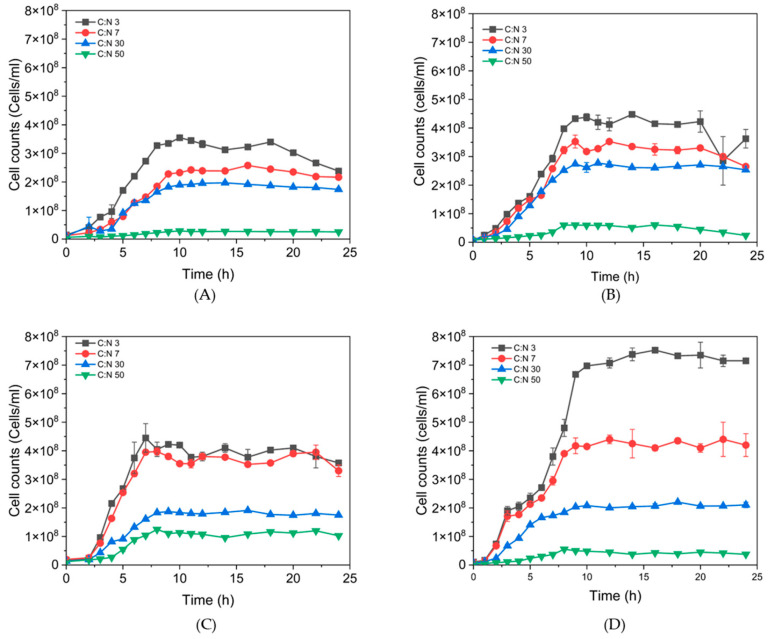
Effect of growth at different C:N ratios of *B. thuringiensis* isolates: (**A**) 32A, (**B**) 42A, (**C**) 73B, (**D**) 81C.

To observe the effects on growth development, Figure 5 shows the maximum cell counts for each isolate at C:N ratios of 3, 7, 30, and 50. It is notable that the observed growth was higher at C:N ratios of 3 and 7, with higher biomass for 81C at C:N 3 ratio. For C:N ratios of 30, the growth was comparable between all tested isolates, while C:N 50 ratio showed the lowest biomass for the four isolates. Additionally, isolates 42A, 73B, and 81C had an inversely proportional growth profile with increasing C:N ratio.

P(3HB) production has been reported in flask fermentations with *Bacillus subtilis,* using cheese whey supplemented with peptone. It exhibited a high P(3HB) concentration at 10% (*v*/*v*) inoculum level, reaching 0.118 g/L of biopolymer. Conversely, using ammonium sulfate and 10% (*v*/*v*) inoculum, an accumulation of 0.096 g/L was obtained, with values similar to those obtained in the present work [28]. P(3HB) is synthesized by diverse microorganisms as an energy storage material under stress conditions, caused by the limited availability of essential nutrients such as nitrogen, oxygen, phosphorus, sulfur, or magnesium in the presence of excess carbon sources [29]. This property can be used to modulate P(3HB) production in bacteria, in particular nitrogen limitation has been reported to lead to P(3HB) accumulation. For instance, Zhou et al. (2022) evaluated the effect of C:N ratios of 1, 10, 40, and 100 using a mixed microbial culture, obtaining weight percentages (wt%) of 3, 26, 52, and 46, respectively, at a pH of 7 [30], thereby, assessing the impact of varying C:N ratios on P(3HB) production. However, in *B. thuringiensis* the information is not concise. Therefore, it was important to evaluate the C:N ratio in the growth and P(3HB) accumulation profiles for the isolates 32A, 42A, 73B, and 81C. The profiles obtained are presented in Figure 5.

**Figure 6:**
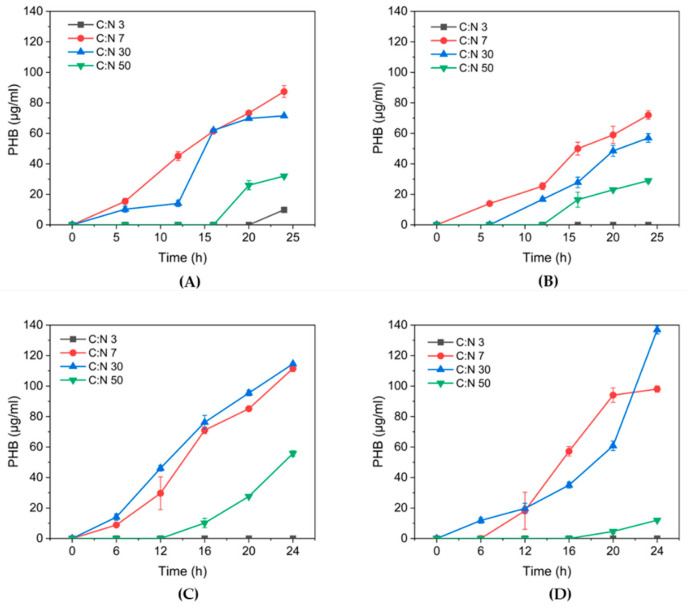
Effect of P(3HB) accumulation at different C:N ratios for isolates (**A**) 32A, (**B**) 42A, (**C**) 73B, (**D**) 81C.

The spectrophotometric calibration curve for P(3HB) determination was described by the regression equation y= 0.0028X + 0.0937 and R^2^ = 0.9891 (Figure S1). The effect of C:N ratio on P(3HB) production was different from that observed on growth (Figure 6). All isolates accumulated P(3HB) at C:N ratios of 7 and 30, but the isolate 81C was the only one that showed the highest amount of P(3HB) (136.9 µg/mL), determined at 30 C:N (Figure 6D). Regarding the C:N 50 ratio, no biopolymer accumulation was detected. This could indicate that a nitrogen deficiency is not essential for biopolymer production, as accumulation is observed with the C:N ratio of 7, that is known as a balanced C:N ratio to produce both spores and Cry protein [21]. P(3HB) production was observed to be undetectable under high carbon and nitrogen limitation conditions, as in our results. Accordingly, Kumar et al. (2015) [3] reported that increasing the nitrogen source content produced significantly higher PHA levels, concluding that as the nitrogen source increases, both bacterial growth and PHA production also increase significantly [31].

**Figure 7:**
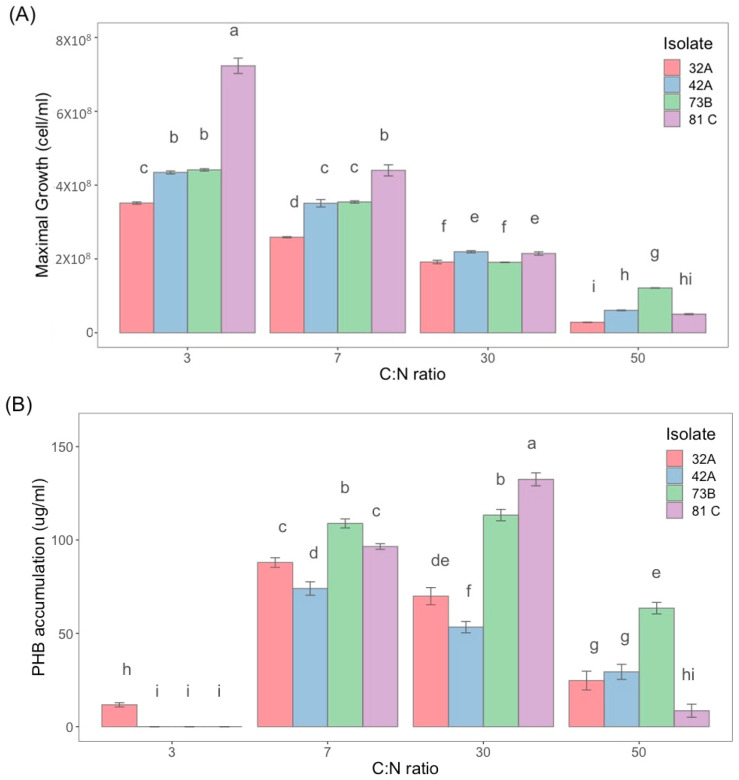
Maximum growth (**A**) and P(3HB) (**B**) accumulation at different C:N ratios in *B. thuringiensis.* Bacterial growth and PHB production were evaluated in different isolates under varying carbon-to-nitrogen (C:N) ratios. Values are expressed as means ± standard deviation (*n* = 3). Statistically significant differences were observed among C:N ratios for both variables (two-way ANOVA, *p* < 0.05), indicating that C:N balance plays a critical role in microbial proliferation and PHB accumulation, regardless of the bacterial isolate. Maximum cell growth was observed at C:N = 3, particularly in isolate 81 C, while significantly lower growth occurred at higher C:N ratios (30 and 50). In contrast, PHB production peaked at intermediate C:N ratios (30 and 7), with markedly reduced or null yields at the extremes (3 and 50). Treatments not sharing the same letter, as determined by Tukey’s HSD post hoc test (*p* < 0.05), differ significantly from each other.

For a better interpretation of the effect of C:N ratio on P(3HB) production, the maximum concentration for each condition and tested isolate is presented in Figure 7. P(3HB) accumulation is observed at both C:N 7 and 30, but the level depends on the Bt isolate. The highest P(3HB) accumulation was shown by 81C with C:N 30 ratio and 2.0 × 10^8^ cells/mL, while at C:N 3 the P(3HB) was not detected, even with 3.8 times the cell counts. For 73B isolate, the P(3HB) concentration was similar at C:N 7 and 30 ratios with 3.3 × 10^8^ and 1.7 × 10^8^ cell counts, respectively. This information suggests that the C:N ratio can differentially affect growth and P(3HB) accumulation in *B. thuringiensis* isolates.

In summary, the best conditions for growth in Bt isolates were C:N 3 and 7, whereas for P(3HB) production, they were C:N 7 and 30. A C:N ratio of 7 was reported previously by Farrera et al. (1998) [32] and Anderson (1990) [33] as the best conditions for growth and Cry protein production. Gowda et al. (2014) [34] reported that with a medium of low nitrogen contents, a maximum growth and therefore a higher PHB yield was obtained in *B. thuringiensis* IAM 12077. In this case, the authors did not report the C:N ratio, but it is estimated that the carbon and nitrogen employed in their reported medium was at a C:N 8 ratio. Conversely, at higher C:N ratios (10 and 20) the P(3HB) formation decreased, despite the fact that it reached a higher biomass concentration, where starch was used as the carbon source and yeast extract as the nitrogen source. On the other hand, Kumar et al. (2015) reported the synthesis of P(3HB) by *B. thuringiensis* strain EGU45 in non-limiting nitrogen conditions using a medium containing crude glycerol and nutrient broth components [31]. Hence, our results correlate with these presentations, suggesting that P(3HB) accumulation can take place at balanced C:N ratios. Otherwise, a higher C:N ratio, such as 50, is not suitable either for growth or P(3HB) accumulation.

**Figure 8:**
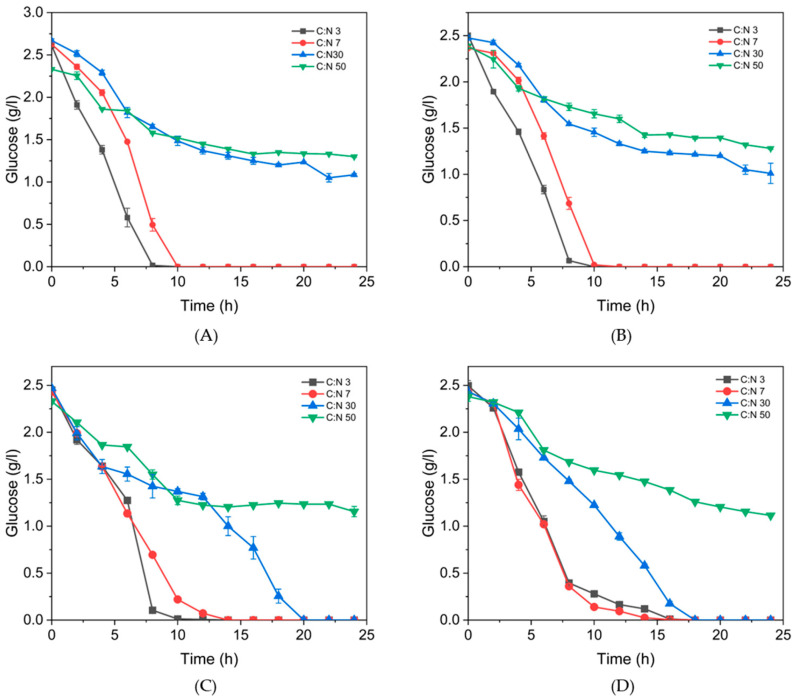
Glucose consumption at different C:N ratios of isolates of *B. thuringiensis.* (**A**) 32A, (**B**) 42A, (**C**) 73B, (**D**) 81C.

To understand the effect of C:N ratio on growth and P(3HB) accumulation, the glucose profiles were analyzed (Figure 8). Glucose was not totally consumed in the 50 C:N ratio for all isolates (Figure 8), whereas the C:N 30 ratio was not depleted for isolates 32A and 42A, but not for isolates 73B and 81C, where the higher accumulation of P(3HB) occurred, even with low cell counts. The carbon source is a major nutritional factor playing an essential role in P(3HB) production, as the bacteria store it in the form of P(3HB) granules [35]. Our information suggests that metabolism of glucose for P(3HB) production can be differentially affected, depending on the isolate requirements and C:N ratio, which is of interest in order to select the ideal *B. thuringiensis* strain, to evaluate in detail the conditions of P(3HB) accumulation. Other studies have concluded that at a high C:N ratio, the accumulation of P(3HB) and bacterial growth are suppressed due to substrate limitation, with elevated glucose concentrations exerting an inhibitory effect, that negatively affects both the specific growth rate and P(3HB) production [36]. Nitrogen limitation does not directly affect acetyl-CoA levels, but by restricting cell growth and creating an excess of available carbon, it favors the accumulation of acetyl-CoA and the subsequent synthesis of PHB [37]. This supports our results at C:N 50 culture which has high carbon availability but lower nitrogen content, and such a condition is not suitable for P(3HB) production with *B. thuringiensis*; the glucose was also not exhausted. In contrast, the presence of high levels of acetyl-CoA within cell compartments is usually associated with an abundance of energy and carbon; such conditions favor anabolism, growth, and cell division [37], but as we observe it depends on the selected C:N ratio.

Table 2 presents the maximum concentrations obtained during flask culture experiments, along with the corresponding calculated yields. Therefore, with this calculation we can corroborate the consistency of the results to select the best C:N ratio and the optimum culture conditions in the bioreactor.

### 3.4. Batch Production of P(3HB) at Bioreactor Scale

**Figure 9:**
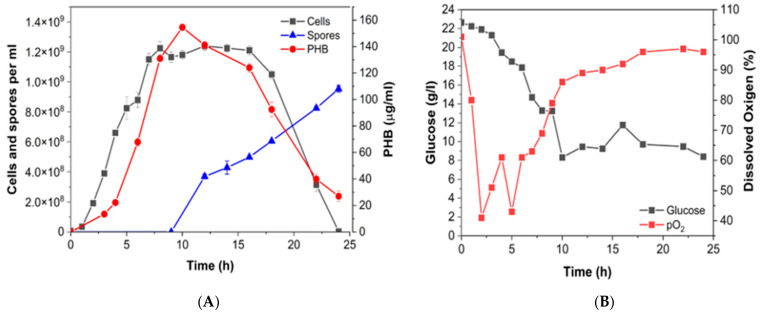
Time course of batch culture for isolate 81C in 7 L reactor with CM at C:N 30 ratio. (**A**) Growth profiles, P(3HB) accumulation, and spore counts; (**B**) glucose consumption and dissolved oxygen.

Based on the above results, isolate 81C was used with a C:N ratio of 30 in CM medium with basal concentration, to produce P(3HB) during the bioreactor cultures (Figure 9). P(3HB) production showed a direct relation with bacterial growth (Figure 9A), reaching a maximum concentration of 152 µg/mL at 10 h, an opposite behavior to that observed in flask cultures where the P(3HB) synthesis was sustained throughout the experiments even in the stationary phase. During exponential growth (0–6 h), the specific growth rate (*μ*) was 1.36 h^−1^ with (R^2^ =0.97). In this case, the maximum cell counts of 1.18 × 10^9^ cells/mL (2.71 g/L cdw) were obtained at 9 h. Glucose consumption was stopped at 10 h with a consumption of 14 g, similar to the behavior determined in flask cultures. Taking this information, we calculated the biomass yield (Y_X/S_) and PHB yield (Y_P/S_) at 10 h, resulting in 0.293 and 0.125, respectively, both different to that observed in the flask culture.

During bioreactor cultures, we observed spores reaching a sporulation efficiency of 76.29%, compared to the flask experiments. Dissolved oxygen (DO) reached low levels of 35% between the transition phase, and then subsequently increased at 90% from 10 h, reaching 96% at 24 h, which describes a characteristic pattern for Bt according to the reports by López and de la Torre [19]. It is noteworthy that glucose consumption finished at 10 h, coinciding with the maximal accumulation of P(3HB), this behavior was different to that observed in flask fermentations at the same C:N ratio. This information suggests that even with the same C:N ratio profiles of growth, P(3HB) accumulation and substrate glucose consumption can be quite different depending on the used culture scale. This explains that for new isolates such as our cellular model, the conditions to develop a bioprocess for biopolymer production require exhaustive characterizations, deep analysis of the results, and optimizations.

Some reports have implemented the use of *Agave durangensis* leaves as a carbon source for P(3HB) production, obtaining 0.34–0.66 g/L with *Bacillus cereus* 4N [38]. The implementation of mixed substrate media incorporating glucose and glycerol has also been employed, resulting in the production of poly(3-hydroxybutyrate-co-3-hydroxyvalerate) at a concentration of 3.57 g/L with Bt during batch fermentations [39]. The P(3HB) obtained in these experiments was used for biopolymer characterization. There are several microorganisms capable of synthesizing P(3HB) polymers, such as the mutant strain *Azotobacter vinelandii* OPNA, which has been reported to achieve a maximal production of 27.3 ± 3.2 g L^−1^ using 60 h fed-batch fermentation, employing the PY sucrose medium [40]. Although the levels of P(3HB) production of Bt are considered low in comparison to other P(3HB) bacterial producers, it is notable that P(3HB) production is growth associated and maximum accumulation occurred entering the stationary phase. This means that P(3HB) production with *B. thuringiensis* has the opportunity to develop further in the near future, where it can be produced in shorter cycles, optimizing labor and energy consumption, which are important issues for large-scale production. Considering the parameters mentioned for batch fermentations, it was possible to increase P(3HB) accumulation using the isolate 81C, whose production increased from 136.9 µg/mL at the flask fermentation to 152 µg/mL in the bioreactor cultures.

### 3.5. Characterization of P(3HB) Extracted from Bt by FTIR

**Figure 10:**
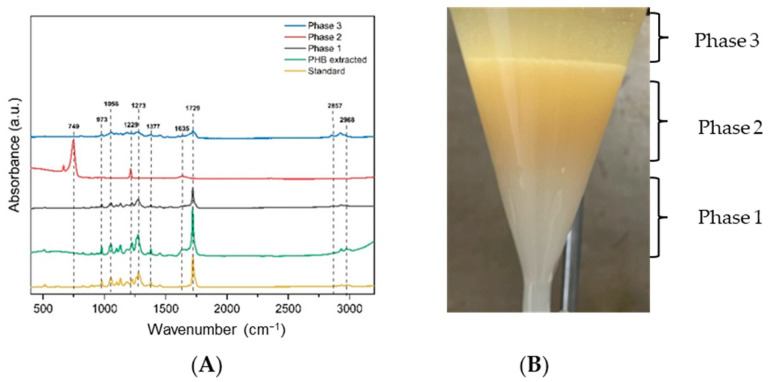
Spectroscopic analysis from P(3HB) extraction. (**A**) Comparison of extraction process by phases by FTIR. (**B**) Different phases of the extraction process.

The extraction from the 24 h fermentation samples is shown in Figure 10, where FTIR characterization was performed for the three different phases to determine whether the phase containing PHB was free of pollutant biomolecules or residual solvents. This will have an influence on the purity of the biopolymer using this extraction method. Phase 1 corresponds to P(3HB) solubilized in chloroform, and the band at 1729 cm^−1^ corresponds to C=O, and could be associated with the P(3HB) present in the sample, also observed in the P(3HB) standard. The bacteria exhibit bands in the range 1539–1651 cm^−1^ associated with peptide components, proteins, and amide groups, as shown for the band at 1642 cm^−1^ in Phase 2, which also corresponds to bending vibrations for fatty acids and proteins [41].

**Figure 11:**
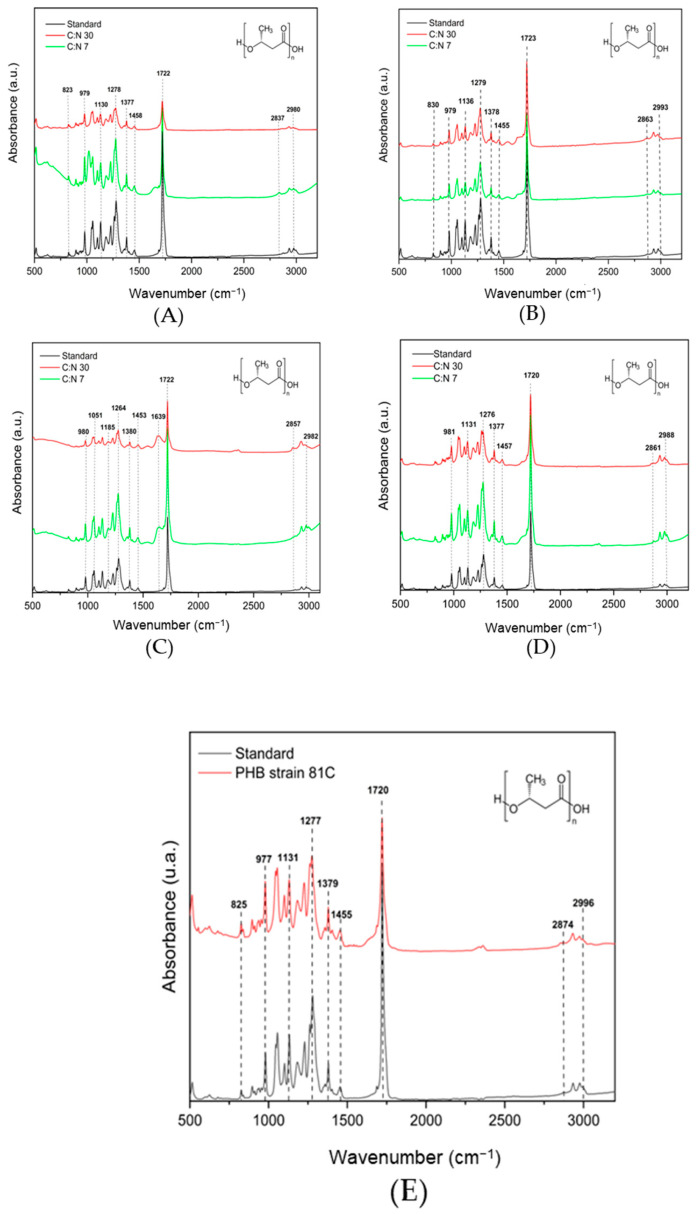
FTIR spectra of P(3HB) produced at 24 h flask cultures by isolates (**A**) 32A, (**B**) 42A, (**C**) 73B, (**D**) 81C, (**E**) P(3HB) extractions from 24 h bioreactor cultures by isolate 81 C.

In this phase, a band is observed at 743 cm^−1^; Naumann et al. referred to the 900–700 cm^−1^ region as the ‘fingerprint region’ because it contains weak yet highly distinctive absorbances that are unique to certain bacteria [42]. Also, bands at 2857 and 2968 cm^−1^ assigned to C–H stretching of methyl and methylene groups are observed in the standard, the P(3HB) extracted, Phase1, and Phase 3 [35]. The extracted P(3HB) was further analyzed by FTIR to determine the characteristic functional groups compared with the P(3HB) standard (Sigma-Aldrich). The FTIR spectra are presented in Figure 11; the bands corresponding to P(3HB) were observed in all isolates with C:N ratios of 30 and 7, where the higher accumulation of the biopolymer occurred. Additionally, P(3HB) was also obtained at flask and reactor level. The P(3HB) fingerprint is located in the 800–1500 cm^−1^ region, indicating the presence of C–CH, C–CH_3_, as well as amorphous (C–O–C) and crystalline (C–C–O) phases [43]. All these bands were determined by the functional groups present in the samples, which were comparable to those presented in the standard. The peak of the carbonyl functional group (C=O) was observed in the range 1725–1725 cm^−1^ [44]. The bands observed in the region 2837–2993 cm^−1^ are associated with the methyl (–CH_3_) and methylene (–CH_2_) groups, respectively [35]. Therefore, the biopolymer obtained from the different Bt isolates is characteristic of the molecular composition of P(3HB).

## 4. Conclusions

Four Gram-positive bacteria isolated from the beach ecosystems of Guerrero were identified through 16S, showing a 100% similarity with *Bacillus thuringiensis*. These bacteria showed biopolymer accumulation depending on the C:N ratio. This work indicates that different C:N ratios used during cultures with Bt affect P(3HB) production, cell growth, and carbon source consumption such as glucose. The obtained results can serve as an important reference for bioprocess development using different raw materials with similar C:N ratios, in distinct cellular models.

The C:N ratios 7 and 30 were considered suitable for biopolymer synthesis in *B. thuringiensis*. Although the 81C isolate accumulates a higher quantity of P(3HB) during bioreactor cultures, this was not significantly higher than that obtained with flask cultures. As we mentioned before, even with the same C:N ratio the growth profiles, P(3HB) accumulation, and glucose consumption can be quite different, and depend on the culture scale used. This is the reason that for new isolates, the conditions to develop bioprocesses to produce biopolymers must be performed and characterized, considering the cellular system, scale, and C:N ratio used. However, the obtained knowledge represents an important step in the development of P(3HB) production, where large-scale production could avoid problems in the purification stage and elaboration of the culture media, as well as benefiting from cost reduction. Likewise, this could establish the basis for implementing Bt as a possible strain for large-scale production, as the main advantage it offers is rapid growth with a maximum biomass production at 10 h, compared to other strains that require up to 48 h or more for growing and accumulating P(3HB).

Future studies focused on gene manipulation and the assessment of nontraditional growth media may enhance output and lower expenses, allowing for the potential use of *B. thuringiensis* in future biopolymer production.

## Figures and Tables

**Table 1:** Media components for setting the different C:N ratios of diluted CM.

C:N Ratio	Glucose (g/L)	Soy Peptone (g/L)	Yeast Extract (g/L)
3	2.5	2.5	4.8
7	2.5	2.5	0.32
30	2.5	0.16	0.20
50	2.5	0.10	0.10

**Table 2:** Maximal biomass produced and P(3HB) yields obtained from flask cultures with *B. thuringinesis* isolates.

Isolate	C:N	Maximal Biomass (g/L)	Y_X/S_	* Y_P/S_
	3	1.73	0.692	Undetectable
	7	1.01	0.411	0.039
81C	30	0.50	0.208	0.056
	50	0.14	0.085	0.008
	3	0.82	0.312	0.004
	7	0.59	0.225	0.034
32A	30	0.45	0.287	0.043
	50	0.07	0.063	0.024
	3	1.02	0.417	Undetectable
	7	0.91	0.376	0.049
73B	30	0.44	0.178	0.046
	50	0.29	0.253	0.049
	3	1.01	0.397	Undetectable
	7	0.81	0.345	0.031
42A	30	0.51	0.203	0.022
	50	0.14	0.057	0.012

* Yields were calculated at the 24 h (maximum P(3HB) determination).

## Data Availability

The original contributions presented in the study are included in the article, further inquiries can be directed to the corresponding author.

## Supplementary Materials

The following supporting information can be downloaded at: https://www.mdpi.com/article/10.3390/polym17141978/s1, Figure S1: Calibration curve for PHB quantification.

## References

[1] Gholamveisi N., Azar S.M., Moravej R. (2018). *Bacillus thuringiensis* Strain NG, a Novel Isolated Strain for Production of Various Polyhydroxyalkanoates. J. Microb. Biol..

[2] Campos M.I., Figueiredo T.V.B., Sousa L.S., Druzian J.I. (2014). The Influence of Crude Glycerin and Nitrogen Concentrations on the Production of PHA by *Cupriavidus necator* Using a Response Surface Methodology and Its Characterizations. Ind. Crop Prod..

[3] Dalsasso R.R., Pavan F.A., Bordignon S.E., de Aragão G.M.F., Poletto P. (2019). Polyhydroxybutyrate (PHB) Production by *Cupriavidus necator* from Sugarcane Vinasse and Molasses as Mixed Substrate. Process Biochem..

[4] Rabnawaz M., Wyman I., Auras R., Cheng S. (2017). A Roadmap towards Green Packaging: The Current Status and Future Outlook for Polyesters in the Packaging Industry. Green Chem..

[5] Reddy M.V., Mawatari Y., Onodera R., Nakamura Y., Yajima Y., Chang Y.-C. (2019). Bacterial Conversion of Waste into Polyhydroxybutyrate (PHB): A New Approach of Bio-Circular Economy for Treating Waste and Energy Generation. Bioresour. Technol. Rep..

[6] Bhatia S.K., Gurav R., Choi T.-R., Jung H.-R., Yang S.-Y., Song H.-S., Jeon J.-M., Kim J.-S., Lee Y.-K., Yang Y.-H. (2019). Poly(3-Hydroxybutyrate-Co-3-Hydroxyhexanoate) Production from Engineered Ralstonia Eutropha Using Synthetic and Anaerobically Digested Food Waste Derived Volatile Fatty Acids. Int. J. Biol. Macromol..

[7] Prasertsilp P., Pattaragulwanit K., Kim B.S., Napathorn S.C. (2023). Microwave-Assisted Cassava Pulp Hydrolysis as Food Waste Biorefinery for Biodegradable Polyhydroxybutyrate Production. Front. Bioeng. Biotechnol..

[8] Jae Park S., Hee Kang K., Lee H., Reum Park A., Eun Yang J., Hoon Oh Y., Keun Song B., Jegal J., Hwan Lee S., Yup Lee S. (2013). Propionyl-CoA Dependent Biosynthesis of 2-Hydroxybutyrate Containing Polyhydroxyalkanoates in Metabolically Engineered *Escherichia coli*. J. Biotechnol..

[9] Reddy C.S.K., Ghai R., Rashmi, Kalia V.C. (2003). Polyhydroxyalkanoates an Overview. Bioresour. Technol..

[10] Kumar P., Patel S.K.S., Lee J.-K., Kalia V.C. (2013). Extending the Limits of Bacillus for Novel Biotechnological Applications. Biotechnol. Adv..

[11] Ielo I., Calabrese G., De Luca G., Conoci S. (2022). Recent Advances in Hydroxyapatite-Based Biocomposites for Bone Tissue Regeneration in Orthopedics. Int. J. Mol. Sci..

[12] Zhang X., Liu X.-Y., Yang H., Chen J.-N., Lin Y., Han S.-Y., Cao Q., Zeng H.-S., Ye J.-W. (2022). A Polyhydroxyalkanoates-Based Carrier Platform of Bioactive Substances for Therapeutic Applications. Front. Bioeng. Biotechnol..

[13] Singh M., Patel S.K., Kalia V.C. (2009). *Bacillus subtilis* as Potential Producer for Polyhydroxyalkanoates. Microb. Cell Fact..

[14] Adnan M., Siddiqui A.J., Ashraf S.A., Snoussi M., Badraoui R., Alreshidi M., Elasbali A.M., Al-Soud W.A., Alharethi S.H., Sachidanandan M. (2022). Polyhydroxybutyrate (PHB)-Based Biodegradable Polymer from Agromyces Indicus: Enhanced Production, Characterization, and Optimization. Polymers.

[15] Zafra G., Absalón Á.E., Cuevas M.D.C., Cortés-Espinosa D.V. (2014). Isolation and Selection of a Highly Tolerant Microbial Consortium with Potential for PAH Biodegradation from Heavy Crude Oil-Contaminated Soils. Water Air Soil Pollut..

[16] Dinorín-Téllez-Girón J., Delgado-Macuil R.J., Larralde Corona C.P., Martínez Montes F.J., De La Torre Martínez M., López-Y-López V.E. (2015). Reactance and Resistance: Main Properties to Follow the Cell Differentiation Process in *Bacillus thuringiensis* by Dielectric Spectroscopy in Real Time. Appl. Microbiol. Biotechnol..

[17] Díaz Pacheco A., Delgado-Macuil R.J., Larralde-Corona C.P., Dinorín-Téllez-Girón J., Martínez Montes F., Martinez Tolibia S.E., López Y López V.E. (2022). Two-Methods Approach to Follow up Biomass by Impedance Spectroscopy: *Bacillus thuringiensis* Fermentations as a Study Model. Appl. Microbiol. Biotechnol..

[18] López Y López V., Mártinez S., Díaz A., Lozano A., Sierra P., Téllez J. (2023). Influence of Nutrient Feeding Variations on AbrB Accumulation, Sporulation and cry1Ac Expression during Fed-Batch Cultures of *Bacillus thuringiensis*. Mex. J. Biotechnol..

[19] López-y-López V.E., De La Torre M. (2005). Redirection of Metabolism during Nutrient Feeding in Fed-Batch Cultures of *Bacillus thuringiensis*. Appl. Microbiol. Biotechnol..

[20] Lozano Goné A.M., Dinorín Téllez Girón J., Jiménez Montejo F.E., Hidalgo-Lara M.E., López Y López V.E. (2014). Behavior of Transition State Regulator AbrB in Batch Cultures of *Bacillus thuringiensis*. Curr. Microbiol..

[21] Monroy M.R., de la Torre M. (1996). Effect of the Dilution Rate on the Biomass Yield of *Bacillus thuringiensis* and Determination of Its Rate Coefficients under Steady-State Conditions. Appl. Microbiol. Biotechnol..

[22] Rajankar M.P., Ravindranathan S., Rajamohanan P.R., Raghunathan A. (2018). Absolute Quantitation of Poly(R)-3-Hydroxybutyric Acid Using Spectrofluorometry in Recombinant *Escherichia coli*. Biol. Methods Protoc..

[23] Singh S., Sithole B., Lekha P., Permaul K., Govinden R. (2021). Optimization of Cultivation Medium and Cyclic Fed-Batch Fermentation Strategy for Enhanced Polyhydroxyalkanoate Production by *Bacillus thuringiensis* Using a Glucose-Rich Hydrolyzate. Bioresour. Bioprocess..

[24] Thammasittirong A., Saechow S., Thammasittirong S.N.-R. (2017). Efficient Polyhydroxybutyrate Production from *Bacillus thuringiensis* Using Sugarcane Juice Substrate. Turk. J. Biol..

[25] Narayanan M., Kandasamy S., Kumarasamy S., Gnanavel K., Ranganathan M., Kandasamy G. (2020). Screening of Polyhydroxybutyrate Producing Indigenous Bacteria from Polluted Lake Soil. Heliyon.

[26] Moorkoth D., Madhavan K. (2016). Production and Characterization of Poly(3-Hydroxy Butyrate-Co-3 Hydroxyvalerate) (PHBV) by a Novel Halotolerant Mangrove Isolate. Bioresour. Technol..

[27] Choi S.Y., Rhie M.N., Kim H.T., Joo J.C., Cho I.J., Son J., Jo S.Y., Sohn Y.J., Baritugo K.-A., Pyo J. (2020). Metabolic Engineering for the Synthesis of Polyesters: A 100-Year Journey from Polyhydroxyalkanoates to Non-Natural Microbial Polyesters. Metab. Eng..

[28] Peña-Jurado E., Pérez-Vega S., Zavala-Díaz De La Serna F.J., Pérez-Reyes I., Gutiérrez-Méndez N., Vazquez-Castillo J., Salmerón I. (2019). Production of Poly (3-Hydroxybutyrate) from a Dairy Industry Wastewater Using Bacillus Subtilis EPAH18: Bioprocess Development and Simulation. Biochem. Eng. J..

[29] Saravanan K., Umesh M., Kathirvel P. (2022). Microbial Polyhydroxyalkanoates (PHAs): A Review on Biosynthesis, Properties, Fermentation Strategies and Its Prospective Applications for Sustainable Future. J. Polym. Environ..

[30] Zhou W., Colpa D.I., Geurkink B., Euverink G.-J.W., Krooneman J. (2022). The Impact of Carbon to Nitrogen Ratios and pH on the Microbial Prevalence and Polyhydroxybutyrate Production Levels Using a Mixed Microbial Starter Culture. Sci. Total Environ..

[31] Kumar P., Ray S., Patel S.K.S., Lee J.-K., Kalia V.C. (2015). Bioconversion of Crude Glycerol to Polyhydroxyalkanoate by *Bacillus thuringiensis* under Non-Limiting Nitrogen Conditions. Int. J. Biol. Macromol..

[32] Farrera R., Pérez F., De La Torre M. (1998). Carbon:Nitrogen Ratio Interacts with Initial Concentration of Total Solids on Insecticidal Crystal Protein and Spore Production in *Bacillus thuringiensis* HD-73. Appl. Microbiol. Biotechnol..

[33] Anderson T. (1990). Efects of Carbon:Nitrogen Ratio and Oxygen on the Growth Kinetics of *Bacillus thuringiensis* and Yield of Bioinsecticidal Crystal Protein. Master’s Thesis.

[34] Gowda V., Shivakumar S. (2014). Agrowaste-Based Polyhydroxyalkanoate (PHA) Production Using Hydrolytic Potential of *Bacillus thuringiensis* IAM 12077. Braz. Arch. Biol. Technol..

[35] Mostafa Y.S., Alrumman S.A., Otaif K.A., Alamri S.A., Mostafa M.S., Sahlabji T. (2020). Production and Characterization of Bioplastic by Polyhydroxybutyrate Accumulating Erythrobacter Aquimaris Isolated from Mangrove Rhizosphere. Molecules.

[36] Patel N., Patel P., Desai R. (2017). Detection & Characterization of PHB (Polyhydroxybutyrate) Producers Halophilic Bacteria Isolated from Marine Water Sample of Valsad District. Int. J. Pharma Bio Sci..

[37] Wang Y., Yang H., Geerts C., Furtos A., Waters P., Cyr D., Wang S., Mitchell G.A. (2022). The Multiple Facets of Acetyl-CoA Metabolism: Energetics, Biosynthesis, Regulation, Acylation and Inborn Errors. Mol. Genet. Metab..

[38] Martínez-Herrera R.E., Alemán-Huerta M.E., Flores-Rodríguez P., Almaguer-Cantú V., Valencia-Vázquez R., Rosas-Flores W., Medrano-Roldán H., Ochoa-Martínez L.A., Rutiaga-Quiñones O.M. (2021). Utilization of Agave Durangensis Leaves by Bacillus Cereus 4N for Polyhydroxybutyrate (PHB) Biosynthesis. Int. J. Biol. Macromol..

[39] Lovely, Kumar S., Srivastava A.K., Shivakumar S. (2024). Optimized Batch Cultivation and Scale-up of *Bacillus thuringiensis* for High-Yield Production of Poly(3-Hydroxybutyrate-Co-3-Hydroxyvalerate). Bioresour. Technol..

[40] García A., Segura D., Espín G., Galindo E., Castillo T., Peña C. (2014). High Production of Poly-β-Hydroxybutyrate (PHB) by an Azotobacter Vinelandii Mutant Altered in PHB Regulation Using a Fed-Batch Fermentation Process. Biochem. Eng. J..

[41] Ghosh S.B., Bhattacharya K., Nayak S., Mukherjee P., Salaskar D., Kale S.P. (2015). Identification of Different Species of Bacillus Isolated from Nisargruna Biogas Plant by FTIR, UV–Vis and NIR Spectroscopy. Spectrochim. Acta Part A Mol. Biomol. Spectrosc..

[42] Naumann D., Helm D., Labischinski H. (1991). Microbiological Characterizations by FT-IR Spectroscopy. Nature.

[43] Martínez-Herrera R.E., Alemán-Huerta M.E., Almaguer-Cantú V., Rosas-Flores W., Martínez-Gómez V.J., Quintero-Zapata I., Rivera G., Rutiaga-Quiñones O.M. (2020). Efficient Recovery of Thermostable Polyhydroxybutyrate (PHB) by a Rapid and Solvent-Free Extraction Protocol Assisted by Ultrasound. Int. J. Biol. Macromol..

[44] Trakunjae C., Boondaeng A., Apiwatanapiwat W., Kosugi A., Arai T., Sudesh K., Vaithanomsat P. (2021). Enhanced Polyhydroxybutyrate (PHB) Production by Newly Isolated Rare Actinomycetes Rhodococcus Sp. Strain BSRT1-1 Using Response Surface Methodology. Sci. Rep..

