# Supplementary figures and images for: Carbon:Nitrogen Ratio Affects Differentially the Poly-β-hydroxybutyrate Synthesis in Bacillus thuringiensis Isolates from México
Source: Polymers (Basel). 2025 Jul 18;17(14):1978. doi: 10.3390/polym17141978 (PMC12299581; doi:10.3390/polym17141978)

## Supplementary material

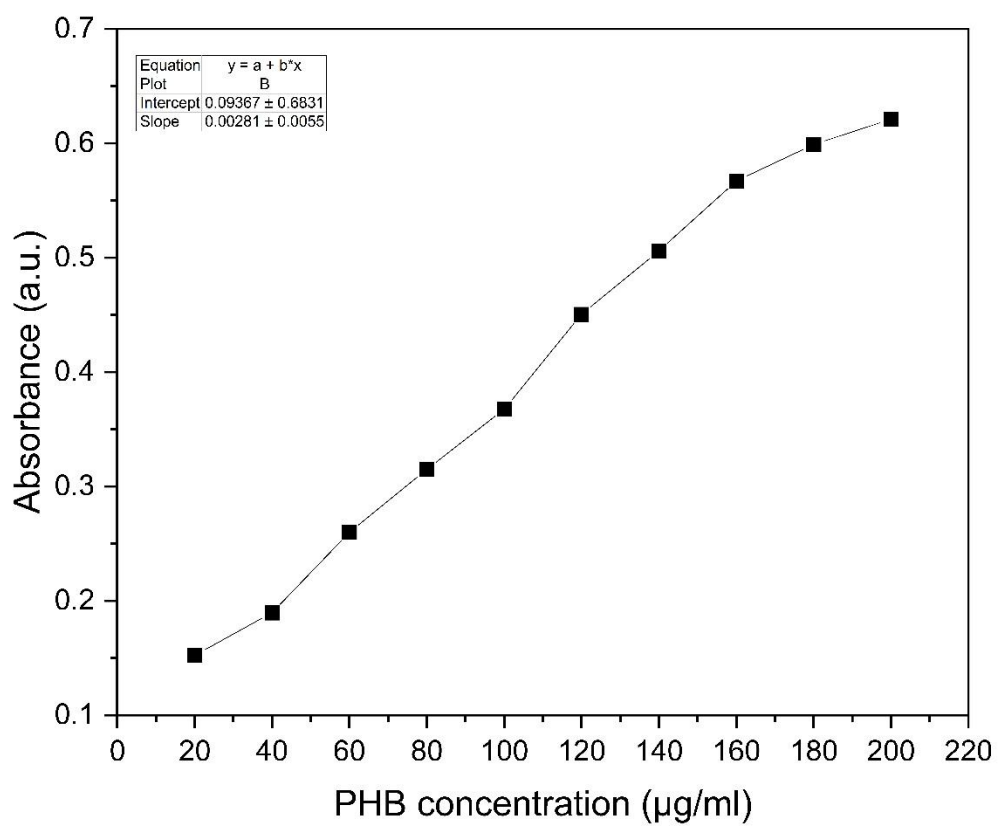

**Figure S1.** Calibration curve for PHB quantification.

Supplement: Supplementary file 1 [file polymers-17-01978-s001.zip › polymers-3689237-supplementary.pdf]
